# Plastics in human diets: development and evaluation of the 24-h Dietary Recall — Plastic Exposure and the Dietary Plastics Score

**DOI:** 10.3389/fnut.2024.1443792

**Published:** 2024-09-18

**Authors:** Amelia Harray, Susan Herrmann, Hannah Papendorf, Claire Miller, Andrea Vermeersch, Tony Smith, Michaela Lucas

**Affiliations:** ^1^Medical School, The University of Western Australia, Nedlands, WA, Australia; ^2^Children’s Diabetes Centre, The Kids Research Institute Australia, Nedlands, WA, Australia; ^3^School of Population Health, Curtin University, Bentley, WA, Australia; ^4^Department of Immunology, PathWest Laboratory Medicine, Nedlands, WA, Australia; ^5^Department of Immunology, Sir Charles Gairdner Hospital, Nedlands, WA, Australia; ^6^Department of Immunology, Perth Children’s Hospital, Nedlands, WA, Australia

**Keywords:** plastic-associated chemicals, dietary intake, dietary assessment, bisphenols, phthalates, 24-h Dietary Recall, plastic exposure

## Abstract

**Background:**

Humans are commonly exposed to plastic through their dietary intake and food consumption patterns. Plastic-associated chemicals (PAC), such as bisphenols and phthalates, are recognized as endocrine-disrupting and are associated with increased risk of cardiovascular disease and metabolic syndrome. However, accurate methods to assess dietary exposure to plastic products and PAC are inadequate, limiting interrogation of health impacts.

**Aim:**

To develop a tool that captures complete dietary exposure to plastics and establish a diet quality score to measure adherence to a low plastic dietary pattern.

**Methods:**

We developed the 24-h Dietary Recall – Plastic Exposure (24DR-PE) and administered it to healthy adults (*n* = 422). This computer-assisted, interviewer-administered tool systematically collects data on food volumes and types, packaging materials, storage, processing, cooking, and consumption methods to assess a food’s exposure to plastic. Specifically, the 24DR-PE incorporates predefined criteria for identifying high-risk practices and food characteristics, such as individually packaged items or those microwaved in plastic, enabling the assignment of scores based on a theoretically derived Dietary Plastics Scoring Matrix.

**Conclusion:**

The 24DR-PE is the first tool specifically designed to capture detailed data on dietary exposures to plastic products. The next step is to validate the score using laboratory results of urine samples we collected contemporaneous to the dietary information. Once validated, the tool has potential for widespread distribution making it valuable for population monitoring, intervention guidance, and future research investigating the interplay between plastics, diet, and human health.

## 1 Introduction

Plastic is a synthetic material manufactured from fossil carbon-based feedstock and comprised of a complex mixture of chemicals. In addition to monomers used to make polymers (such as Bisphenol-A), there are a wide range of other chemical additives, for example phthalate plasticisers (1). Many additives are not covalently bonded to the polymer and leach out (2) and unreacted monomers are also released (1). Plastic is light, malleable, convenient, and used extensively in construction, commercial and consumer materials, including food products (3). Unsurprisingly, dietary intake is a major source of human exposure (4–6), because plastic is used extensively in the processing, packaging, preparation, cooking and consumption of food.

As well as through ingestion, plastic chemicals enter the body via inhalation of air and dust, and absorption through the skin (7). Physiologically, plastic chemicals interfere with hormonal function and have been associated with an increased risk of developing metabolic syndrome, cardiovascular disease, and other chronic health conditions (8–12). Despite extensive exposure of humans to plastic products, as evidenced by detection in urine, serum, nasal secretions, semen, adipose, and brain tissue (13–18), a comprehensive assessment method to quantify individual dietary plastic exposure is lacking (4). Such a tool could provide the necessary discrimination required to explore causal relationships with health outcomes, guide dietary interventions aimed at minimizing exposure to endocrine disrupting chemicals, and inform food regulatory practices.

Within households, plastic chemicals can leach into food from packaging materials, food preparation, cooking methods, appliances and utensils. Certain characteristics of food appear to lead to higher concentrations of chemical additives, including those with acidic, aqueous, fatty and alcoholic properties. In addition, there are exposure risks inherent in food that is meat-based, canned, highly processed, individually packaged, and microwaved in plastic (19). Animals consumed by humans are exposed to plastic additives and as such, are an intrinsic source of a wide range of synthetic chemicals, including those that disrupt the endocrine system (20). While regulatory measures to minimize human dietary exposure to plastic chemicals vary between countries, they most commonly relate to Bisphenol A. Recently, less researched and regulated substitutes, such as Bisphenol S, are now used and are not deemed a safer alternative (21–23).

While earlier studies have measured dietary plastic product exposure using paper-based and electronic methods, such as modified food frequency questionnaires (FFQ), 24-h recalls (24HR), and semi-quantitative questionnaires (24–36), there is no comprehensive tool available to assess potential plastic exposures from the point of food purchase to consumption. Kataria et al. (31) conducted telephone interviews with a self-administered questionnaire that gathered information on food packaging and preparation. Meanwhile, Casas et al. (33) included additional questions in an FFQ about water volume and type, organic food consumption, plastic microwave and food container use and foods packaged in plastic or cans. Another study asked participants to record packaging and cookware materials and the use of thermal paper in a 24-h weighed food record (36). However, the dietary assessment tools used in these studies lacked critical information concerning the duration of food heating (a known high-risk transfer method), microwave power settings, the freezing and thawing of foods (known to increase plastic chemical transfer), food decanting and storage practices and materials, or the use of plastic crockery and cutlery when eating (37).

An important factor missing in earlier studies has been the lack of contemporaneous biological samples obtained from the same period dietary intake was measured. Urinary concentrations of bisphenols and phthalate metabolites are proxy measures of plastic exposure, but the half-life of these chemicals is relatively short (38). Therefore, both the timing of biological samples and the collection of dietary intake data should be during the same time frame. Without confirmatory urinary excretion data, the validity of a dietary assessment tool to quantify plastic exposure cannot be established.

Best practice in dietary assessment is to modify an existing and suitably validated tool to answer a research question (39). We chose to modify the validated 24-h automated multiple pass method (AMPM) widely used in nutritional epidemiology (40). This method has been used in a healthy adult population and validated against doubly labeled water in adults (41). This approach involves obtaining detailed dietary intake data using a five-step process, allowing for more than one ‘pass’ in questioning in order to record additional detail (42, 43). The AMPM has been adapted as the online Automated Self-administered 24-h recall (ASA24) by the National Cancer Institute (44).

Conducting multiple 24-h recalls can estimate ‘usual intake’ (45, 46) and interviewer-administered recalls have higher reporting accuracy (47). Compared with FFQ, 24-h recalls are not limited by a set number of foods, rely less on long-term memory, collect pertinent information on dietary practices, and, in the case of plastics, can collect dietary intake data for the specific time biological samples are obtained.

This manuscript describes (1) the design and development of the 24-h Dietary Recall – Plastic Exposure (24DR-PE) tool to capture individuals’ dietary exposure to plastic, and (2) the development of a diet quality score to measure adherence to a low plastic dietary pattern.

## 2 Materials and equipment

### 2.1 Computer-assisted data collection software

The research team, comprising of clinical researchers with postgraduate qualifications in nutrition and dietetics, worked with an information technology specialist to design a Research Electronic Data Capture (REDCap) program to comprehensively capture plastic product exposure via an interview process. The REDCap software program is a secure web platform used widely for building and managing online surveys and databases, and enables the dissemination of tools globally (48, 49).

### 2.2 Food model booklet

Paper copies of the Australian Bureau of Statistics (ABS) Food Model Booklet were provided to participants to assist in estimating food and beverage portion sizes during the dietary interviews (50). The booklet contains images and figures that represent different portion sizes. Visual representations included wedges, slices, mugs, meat portions, glasses and takeaway containers. The reference code for each Food Model Booklet image was a drop-down option in the 24DR-PE database.

## 3 Methods

### 3.1 Objective

The objectives of this study were to (1) review the literature to identify plastic exposures from food purchase to consumption, (2) develop a computer assisted 24-h Dietary Recall – Plastic Exposure (24DR-PE) tool using REDCap on a data collection platform, and (3) derive a Dietary Plastics Scoring Matrix to summarize high risk plastic foods into a Dietary Plastics Score to measure adherence to a low plastic dietary pattern.

### 3.2 Development and evaluation of the 24DR-PE method

**Figure 1:**
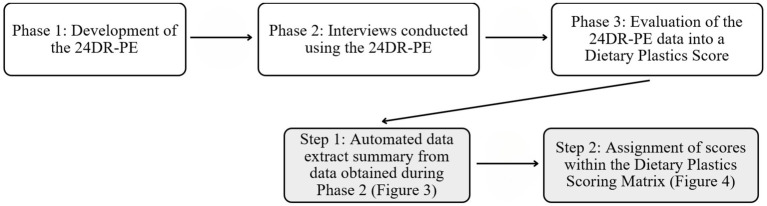
Phases of the development, application and evaluation of the 24DR-PE.

The development and evaluation of the 24-h Dietary Recall – Plastic Exposure and Dietary Plastics Score took part in three phases, with the evaluation involving two steps (see Figure 1).

#### 3.2.1 Phase 1: development of 24DR-PE

The development of the 24DR-PE was a multistage process, commencing with a review of the literature, followed by a review of available dietary assessment methods that could be modified to assess plastic exposures, along with food and nutrient intake and the timing of eating occasions. Weekly team meetings were held to discuss directions provided in the literature, dietary practices within the home, and actively build the data collection tool – the 24DR-PE.

The 24-h dietary recall automated multiple pass method (AMPM) was selected as the most appropriate base method to gather details on dietary plastic exposure at an ingredient level, as it is a previously validated and widely used method for identifying food and nutrient consumption. The choice of a 24-h recall method allowed for the assessment of the same 24-h period during which biological samples were being collected, making a comparison between biological sampling and the 24DR-PE possible. The developers of the AMPM tool were contacted, but it was deemed unfeasible to directly modify it to the level of detail required to capture dietary plastic exposures for our study. We therefore developed our own computer-assisted tool based on the AMPM, which allowed for a standardized stepwise process, incorporating pre-determined options, in-built branching and skip logic.

Nutrition analysis software was not incorporated into this database as the software investigated was not capable of being automated to run within the REDCap database system (51). As such, the database, including food brands, specific ingredients, and amounts consumed, was formatted to allow for easy export into nutrition analysis software. In this way, nutrition and energy intake can be calculated from the data obtained through the 24DR-PE.

A 24DR-PE User Manual was written and reviewed by the research team prior to participant recruitment. Testing of the 24DR-PE was carried out by team members, with the support of two Master of Dietetic research students. To evaluate its usability, a series of pilot tests were undertaken in a duplicate training database to identify potential technical issues and ambiguities in the tool’s questions, prompts and pace.

#### 3.2.2 Phase 2: 24DR-PE interviews with participants

The 24DR-PE interviews were conducted with 211 healthy adults, aged 18–60 years, who participated in the Plastic Exposure Reduction Transforms Health (PERTH) Trial in Perth, Western Australia. Recall interviews were undertaken with each participant on two occasions, within 1 week. The first was conducted in person and captured the preceding weekday, while the second was conducted via telephone on a Monday to capture dietary intake on a weekend day. Trained Accredited Practising Dietitians conducted each interview using a five-step process (outlined below) taking between 45 and 60 min to complete. The length was dependent on the number of food items consumed.

##### 3.2.2.1 Step 1: quick list

The purpose of the ‘quick’ list was to obtain a recall of all foods, beverages, supplements, and medications consumed in the preceding 24 h, from midnight to midnight. Food items and brand names were entered in free-text fields because a pre-determined list of foods was considered too restrictive given the broad scale of available foods and beverages in Australia.

Data collected during this step included a question about whether the preceding 24-h was considered a ‘typical day of intake,’ and details on water intake, such as the volume, the source and drinking vessel. Sources of water were tap, filtered from tap, filtered in plastic jug, rainwater from a tank, bore water, plastic bottled water and other. Drinking vessels included glass, ceramic mug, plastic cup, plastic bottle, metal bottle and other. The measure excluded water in tea, coffee and other beverages consumed, which were captured subsequently in Step 4 of the 24DR-PE.

##### 3.2.2.2 Step 2: forgotten foods list and additions

Dietitians asked participants if they consumed any other items, or any foods from a list of commonly forgotten foods. These items were added and allocated to a separate or pre-completed eating occasion.

##### 3.2.2.3 Step 3: time and eating occasion

The time of each eating occasion was recorded in a 24-h format. Eating occasions were classified as a time in which foods, beverages or supplements were consumed within one sitting. This was considered important to ensure plastic dietary exposure measures can be validated against biological samples obtained during the same 24-h period.

##### 3.2.2.4 Step 4: detailed cycle

**Figure 2:**
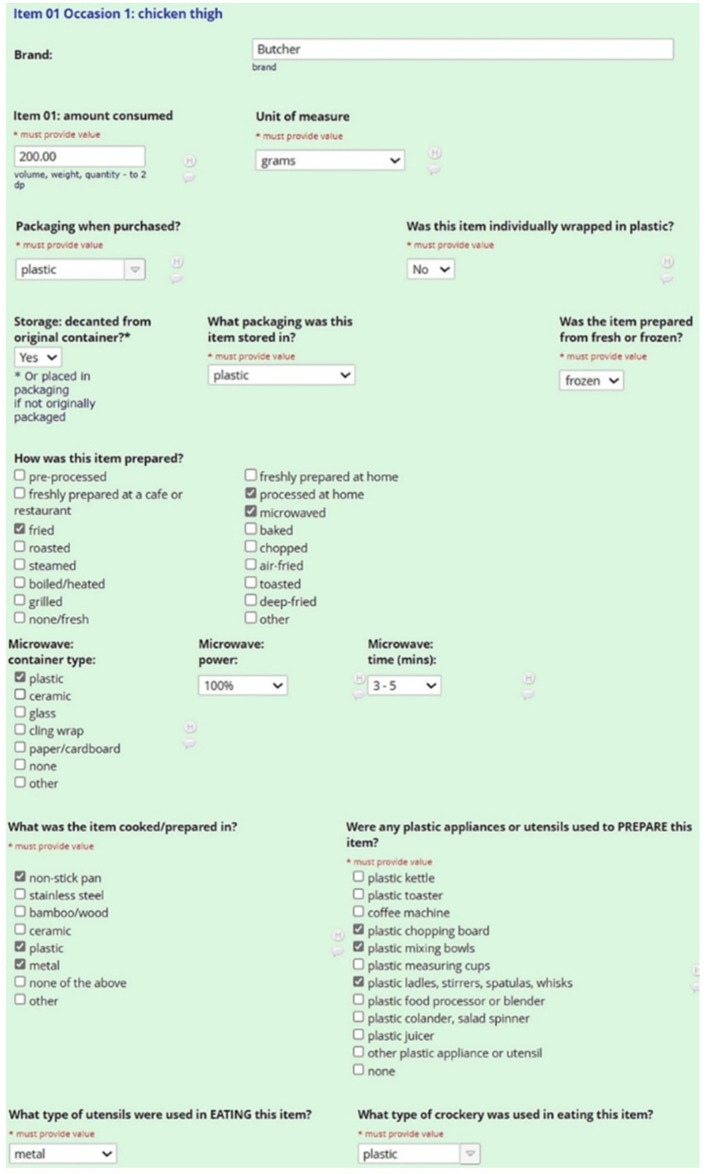
Example of a single food item captured during the 24DR-PE interview.

Step 4 collected descriptive information about each ingredient or item consumed, including brand names and volumes. If participants struggled to estimate portion sizes or volumes, they were encouraged to refer to the hard-copy ABS Food Model Booklet and report the most relevant portion size and corresponding code. The interviewer then selected the booklet code from pre-defined dropdown options, which automatically calculated the weight volume. For each food item or ingredient, dietitians used standardized prompts to capture detail on potential plastic exposures from food packaging, storage, preparation and consumption (see Figure 2).

###### 3.2.2.4.1 Food packaging

Participants were asked about the packaging of food or ingredient purchased. Options included plastic, cardboard, tin (can), polystyrene, paper, cloth, glass, stainless steel, bamboo/wood, ceramic, foil, none (loose) and other. This was followed by a separate question asking if the item was individually packaged. The rationale for this was due to the increased surface area of food touching plastic when individually packaged. If a food was purchased or prepared by someone else and the participant did not know this level of detail, there was on option to specify this in the recall stage.

###### 3.2.2.4.2 Storage

Food may be transferred or decanted into a plastic or non-plastic material after purchase, so participants were asked if the food was kept in its original packaging. If the response was ‘yes,’ details were obtained on the storage material. The options provided were plastic, cardboard, tin (can), polystyrene, paper, cloth, glass, stainless steel, bamboo/wood, ceramic, foil, none (loose) and other.

###### 3.2.2.4.3 Preparation

Because defrosting foods can enhance the transfer of plastic chemicals into food, we asked participants if ingredients were fresh or frozen (37). The materials of utensils and appliances involved in the preparation of foods were recorded, such as chopping, grating, blending or slicing. Options for plastic appliances and/or utensils used included plastic kettles, toasters, chopping boards, bowls, cups, ladles/stirrers, blenders, colanders, juicer, coffee machines or other.

###### 3.2.2.4.4 Cooking

We captured information about the material of any appliances or utensils used in heating and cooking each food item, for example non-stick cookware, metal, bamboo/wood, stainless steel, plastic, metal or other. We also recorded cooking methods including boiling, baking, frying, steaming, roasting or processed in any other way. The latter option allowed the interviewer to enter free text. If a microwave was used to heat food, details on the material of the vessel used was captured. If food was microwaved in plastic, additional questions were asked to capture details on the heat setting and the length of time it was heated. All additional questions were incorporated as branching logic to ensure complete information capture.

###### 3.2.2.4.5 Consumption

The 24DR-PE captured volumes consumed in grams, cups, milliliters or items (e.g., one medium banana), the brands of foods, and the materials of the crockery and cutlery involved in consuming each item. Examples of material options for crockery and cutlery included plastic, cardboard, polystyrene, metal, bamboo/wood, ceramic, glass, tin and other.

##### 3.2.2.5 Step 5: final probe

Participants were asked to reflect and recall missed foods, beverages, supplements or medications consumed, or add other information.

#### 3.2.3 Phase 3: data evaluation

**Figure 3:**
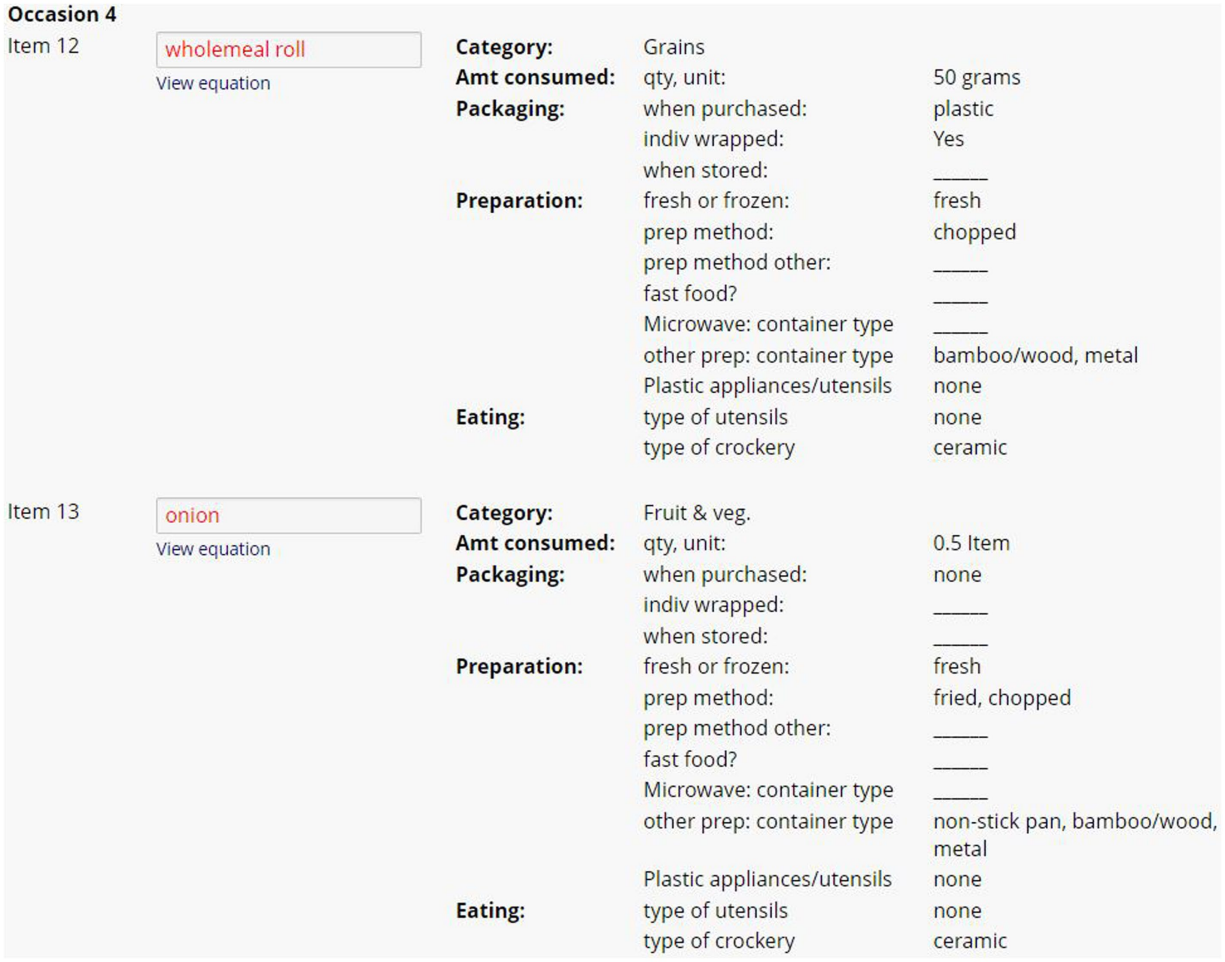
Example of summary of dietary plastic exposure data from two ingredients within one eating occasion in the 24DR-PE. This figure provides an overview of the dietary plastic intake data collected from the assessment, highlighting high-risk plastic exposure events.

**Figure 4:**
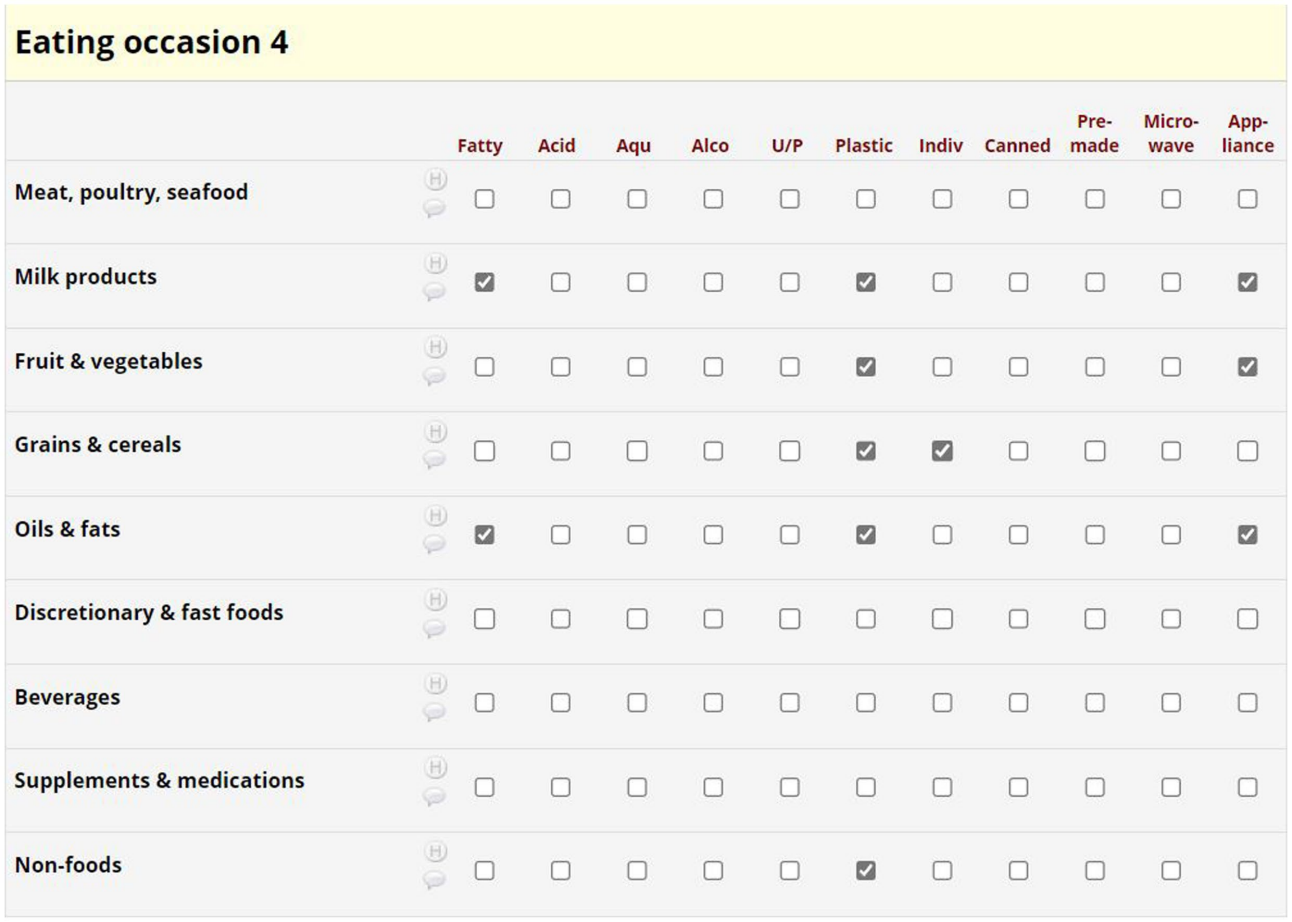
Example of the Dietary Plastics Scoring Matrix.

Evaluation of 24DR-PE data involved two steps (Figure 1). The interview data was automated into a summary of high-risk plastic exposures (Figure 3), and the manual completion of the Dietary Plastics Scoring Matrix to determine a Dietary Plastics Score (Figure 4).

##### 3.2.3.1 Automated summary of plastic exposures

At the conclusion of each interview, the program automatically converted commonly reported participant measurements (i.e., tsp., mL, cups etc.) and ABS Food Model Booklet measurements into gram weights. The 24DR-PE was pre-programmed to extract selected data, as determined by the research. The data extract provided an overview of the amount of each item consumed, and the material used in food packing, storage, preparation and consumption. Items were organized chronologically by eating occasion (Figure 3). The dietitians used this to assign binary scores into the theoretically derived Dietary Plastics Scoring Matrix (Figure 4).

##### 3.2.3.2 Determining a Dietary Plastics Score

We developed a Dietary Plastics Scoring Matrix to determine an individual’s Dietary Plastics Score. Diet quality indices are used to summarize dietary patterns and adherence to a certain dietary pattern, in our case, dietary practices that are low in plastic. Our putative Dietary Plastics Scoring Matrix (Figure 4) is a binary scoring system developed using existing literature on high-risk foods (e.g., discretionary and fast foods, acidic, and aqueous) and behaviors (e.g., use of canned food or microwaving in plastic).

Foods were categorized into the Australian Dietary Guidelines food groups (*y*-axis of Figure 4), with separate categorizations for nutritional supplements, non-foods and beverages (52). High risk plastic elements in the Dietary Plastics Scoring Matrix (*x*-axis of Figure 4) included whether the food item was aqueous, alcoholic, acidic, fatty, ultra-processed [based on the NOVA classification system (53)], wrapped in plastic packaging, individually wrapped, premade, microwaved in plastic, or involved plastic appliances or equipment in its preparation or consumption (Figure 3).

A dietitian used the automatically generated data extract (Figure 3) to complete the Dietary Plastics Scoring Matrix (Figure 4). Each food item within an eating occasion received a categorical check if they satisfied the criteria, for instance, consuming an individually packaged chocolate bar as a snack was categorized under the food group ‘discretionary and fast food’ with checks in fatty, ultra-processed, plastic, and individually packaged rows. If multiple food groups within each eating occasion satisfied the same criteria, further checks were not added. All categorical checks carried equal weight, and the overall number of checks were summarized to estimate the risk of plastic exposure for each eating occasion. There were no limits to the number of eating occasions or food items in the 24-h period, hence there is no maximum Dietary Plastics Score. However, a lower score indicates lower dietary plastic exposure.

##### 3.2.3.3 Inter-rater reliability

We conducted inter-rater reliability on 20% of the collected 24DR-PE. Using an online random integer generator (54), we randomly selected unique participant identification codes. If the selected ID integer did not have an associated 24DR-PE, we chose the next number in numerical order. We re-evaluated both the initial and telephone 24DR-PE for each Participant ID, categorizing them as one ‘case’.

One of the dietitians, who did not fill out the original Dietary Plastics Scoring Matrix, independently re-evaluated the same 24DR-PE summary on selected participants. Each re-evaluation involved completing a separate Dietary Plastics Scoring Matrix template, created in Microsoft Excel, and comparing it to the original scoring allocation. Commonalities and discrepancies between dietitians were identified and discussed at team meetings to reach a consensus. These decisions were documented and informed subsequent Matrix scoring decisions.

## 4 Discussion

This manuscript outlines the design, development, and evaluation of the 24-h Dietary Recall – Plastic Exposure (24DR-PE) tool and the associated Dietary Plastics Scoring Matrix. We developed and administered the computer-assisted 24DR-PE to obtain comprehensive data on individuals’ dietary plastic exposure, providing the most detailed dietary assessment tool available in the literature. This tool will help further investigate the relationship between plastic exposure, diet, and human health.

Previous methods for exploring the relationship between diet and plastics have not measured plastic exposure in such detail and have not collected urinary plastic metabolite data contemporaneously (24–36). Currently, no validated dietary assessment tool exists to assess dietary plastic exposures, nor is there a diet quality index to measure adherence to a low-plastic dietary pattern. Given the health and environmental impacts of plastics (55) and the public interest in reducing plastic use, a personal Dietary Plastics Score is a timely and valuable contribution to the field.

In developing the 24DR-PE, our interdisciplinary team of researchers, dietitians, and a database specialist considered all aspects of food packaging and consumer dietary practices. We integrated this knowledge into a user-friendly online platform using a stepwise approach to enhance data completeness and accuracy (56–59). The development of the 24DR-PE adhered to best practice guidelines by modifying an existing validated tool, which was then administered by trained dietitians to healthy adults. This method improves accuracy in estimating portion sizes and minimizes the likelihood of missing data compared to self-administered recalls (60). However, exploring self-administration of the 24DR-PE using REDCap could reduce researcher burden.

The 24DR-PE could be further refined to incorporate image-assisted recall data, reducing recall burden and improving food identification and portion size estimation (61, 62). Image-assisted recalls have previously been used to assess individually packaged foods (62) and may also provide more accurate estimates of energy and macronutrient intake compared to traditional interviewer-administered 24-h recalls (61).

We conducted two 24DR-PE interviews for each participant (*n* = 422 recalls) to estimate usual dietary intake and reduce random error. The iterative process used supported multiple repeated measures, enhancing our ability to observe trends and variations in plastic exposure and dietary behavior over time, both within and between individuals (63, 64).

To summarize and synthesize the detailed data obtained during the 24DR-PE interviews, we pre-programmed an automatic extraction of high-risk plastic behaviors (e.g., microwaving fatty foods in plastic). A trained interviewer then used this data to assign scores for each eating occasion using our Dietary Plastics Scoring Matrix. This scoring system assesses adherence to a low-plastic dietary pattern and generates an overall Dietary Plastics Score for each 24DR-PE completed. The development, evaluation, and refinement of our Dietary Plastics Score as evidence on the health impacts of plastics becomes available, will facilitate population-based monitoring and enable individuals to predict their dietary plastic exposure.

During the PERTH Trial, participants collected three urine specimens within 24-h (morning, afternoon, and bedtime), labeling and freezing them immediately. This urine collection coincided with the dietary intake capture period. We plan to validate our Dietary Plastics Score against these urinary metabolites (*n* = 422). Urinary metabolites consisted of the bisphenols BPA, BPS, BPF, BP-AF, BPB, BPZ, BP-AP, and the metabolic products of the phthalates Diethyl phthalate (DEP), Di-iso-butyl phthalate (DIBP), Di-(2-ethyl-hexyl) phthalate (DEHP), Di-n-butyl phthalate (DnBP), Di-isononyl phthalate (DINP), Butyl benzyl phthalate (BBP), Dimethyl phthalate (DMP), Di-isononyl phthalate (DINP). As the chemical make-up of plastics varies significantly between products, the Dietary Plastics Score was used to estimate the overall risk of plastic exposure. As such, validation will consist of correlating the score with each urinary metabolite, as well as with the aggregate exposure level. Additionally, stool samples collected and stored during the trial will be analyzed for micro and nano-plastics to further validate the tool. Immature methods to accurately assess nano- and microplastics and the high cost of such assessments have precluded us doing this in the current study.

A limitation of the 24DR-PE is the recall length of 45–60 min. This increased participant and researcher burden and may have led participants to simplify their dietary practices or intake for their second recall. An inherent limitation in all retrospective dietary assessment methods is the reliance on memory and the potential for recall and social desirability biases (65, 66). To reduce this, our team of Accredited Practising Dietitians built rapport with participants and requested that they not alter their behavior between recalls.

Another consideration in this emerging area of dietary assessment is that participants may unintentionally misreport food packaging and cooking materials due to lack of awareness or the deceptive nature of plastic packaging, such as plastic-lined cardboard coffee cups or coated cast-iron cookware (67). Additionally, capturing plastic exposure from foods prepared outside the home, such as a sandwich from a café, is challenging without testing the food itself. As Australians dine out two to three times per week (68), it is challenging to minimize this limitation. The Dietary Plastics Scoring Matrix considered pre-made food as a high-risk dietary behavior.

## 5 Conclusion

The development of the 24-h Dietary Recall – Plastic Exposure tool, and the Dietary Plastics Scoring Matrix represents a significant advancement in assessing complex dietary plastic exposures providing a level of detail not previously obtained. As evidence of the health and environmental impacts of plastic grows, the need for quantitative methods to accurately measure dietary plastic exposure will increase. The use of REDCap allows the 24DR-PE to be widely distributed to researchers along with an instruction manual. The dissemination potential of the 24DR-PE will support research into the health impacts of ingested plastic, facilitate population monitoring of dietary plastic exposures, guide dietary recommendations, and inform policy changes aimed at reducing plastic use for health and environmental reasons.

## Data Availability

The raw data supporting the conclusions of this article will be made available by the authors, without undue reservation.
